# Tailored Mass Spectral Data Exploration Using the SpecXplore Interactive Dashboard

**DOI:** 10.1021/acs.analchem.3c04444

**Published:** 2024-04-02

**Authors:** Kevin Mildau, Henry Ehlers, Ian Oesterle, Manuel Pristner, Benedikt Warth, Maria Doppler, Christoph Bueschl, Jürgen Zanghellini, Justin J. J. van der Hooft

**Affiliations:** † Department of Analytical Chemistry, University of Vienna, 1090 Vienna, Austria; ‡ Austrian Centre of Industrial Biotechnology (ACIB GmbH), 8010 Graz, Austria; ( Doctoral School in Chemistry, University of Vienna, 1090 Vienna, Austria; ∥ Institute of Visual Computing and Human-Centered Technology, TU Wien, 1040 Vienna, Austria; ⊥ Department of Food Chemistry and Toxicology, University of Vienna, 1090 Vienna, Austria; # Department of Biophysical Chemistry, University of Vienna, 1090 Vienna, Austria; ¶ University of Natural Resources and Life Sciences (BOKU), 3430 Tulln, Austria; ∇ Bioinformatics Group, Wageningen University, 6708PB Wageningen, The Netherlands; ○ Department of Biochemistry, University of Johannesburg, 2006 Johannesburg, South Africa

## Abstract

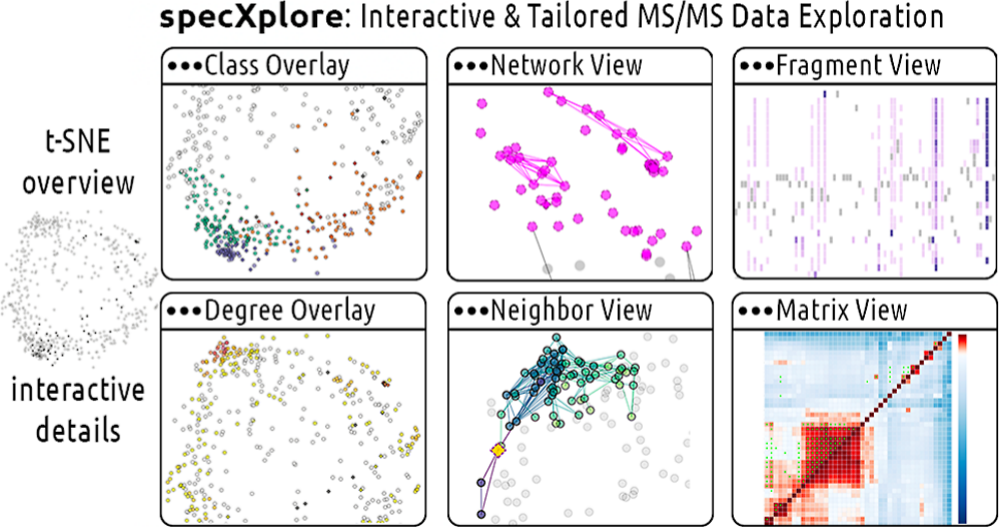

Untargeted metabolomics promises comprehensive characterization of small molecules in biological samples. However, the field is hampered by low annotation rates and abstract spectral data. Despite recent advances in computational metabolomics, manual annotations and manual confirmation of in-silico annotations remain important in the field. Here, exploratory data analysis methods for mass spectral data provide overviews, prioritization, and structural hypothesis starting points to researchers facing large quantities of spectral data. In this research, we propose a fluid means of dealing with mass spectral data using specXplore, an interactive Python dashboard providing interactive and complementary visualizations facilitating mass spectral similarity matrix exploration. Specifically, specXplore provides a two-dimensional t-distributed stochastic neighbor embedding embedding as a jumping board for local connectivity exploration using complementary interactive visualizations in the form of partial network drawings, similarity heatmaps, and fragmentation overview maps. SpecXplore makes use of state-of-the-art ms2deepscore pairwise spectral similarities as a quantitative backbone while allowing fast changes of threshold and connectivity limitation settings, providing flexibility in adjusting settings to suit the localized node environment being explored. We believe that specXplore can become an integral part of mass spectral data exploration efforts and assist users in the generation of structural hypotheses for compounds of interest.

## Introduction

Untargeted metabolomics deals with the elucidation and characterization of small molecules in complex biological systems. Small molecules or metabolites cover an enormous chemical diversity involved in a vast range of biological functions. This chemical diversity leads to complex and heterogeneous data that is difficult to provide consistent and automated workflows for.^1^ Computational metabolomics tools which assist manual data evaluation and annotations efforts such as experimental networking thus remain critical to the field.^2^ Molecular Networking (MN) hosted on the Global Natural Products Social Molecular Networking (GNPS) servers is possibly the most used computational metabolomics tool for exploratory data analysis work using liquid chromatography tandem mass spectrometry (LC–MS/MS) data.^3−6^ The core idea behind MN is that, since similar structures tend to fragment similarly, spectral similarity may be used to construct spectral feature groups with implied structural similarity. In MN, the modified cosine score similarity matrix of the measured spectra forms the basis for constructing such groups using a network topology approach.^3^ The nodes in the network represent MS/MS spectral features that may be connected via edges as a function of pairwise spectral similarity. Indeed, pairwise similarity thresholds and other network processing parameters are used to filter the complete network of all possible pairwise connections such that only edges for high pairwise spectral similarities remain. Using this approach, interrelated spectra are used to form small, separated (disjoint) groups of nodes of high intraspectral similarity commonly referred to as molecular families.^3^ MN can thus be viewed as an exploratory analysis framework merging topological grouping and network visualization. Molecular families serve two separate functions: (a) they provided an ordered data overview and (b) they may be used to assist network annotation propagation, that is, the propagation of structural hypotheses from known structures to unknown ones via proximity in the network.^5,7−9^

While the MN workflow is hugely successful, it comes with its own trade-offs.^5^ For instance, the use of the modified cosine score and minimum fragment overlap requirements poses a stringent similarity criterion for connectivity, resulting in rather sparse networks suitable for representation as disjoint subnetworks. However, the modified cosine score may miss structurally related analogues which exhibit larger fragmentation differences, while the disjointness of the molecular families may obscure relationships between groups. Importantly, MN operates using a single global threshold setting on the basis of which connectivity may or may not exist. Such a global threshold is unlikely to work well for all chemical families measured, where some may exhibit much richer or much sparser fragmentation or overlap thereof. In addition, the combination of disjointness of spectral groupings, as well as the disconnected runs with new randomly generated layouts for each molecular family make setting comparisons an arduous task.

In this paper, we introduce specXplore, an interactive Python dashboard aimed at facilitating spectral data exploration in a flexible and local network topology tailored fashion. Unlike traditional molecular networking, specXplore was created with adjustable settings for heterogeneous and dense network data in mind. SpecXplore provides complementary and interactive visualizations that allow users to explore connections between the spectral features using interactively adjustable network settings.

SpecXplore consists of an importing module providing data integration capacities and a dashboard-module for interactive exploratory data analysis. The dashboard makes use of a two-dimensional t-distributed stochastic neighbor embedding (t-SNE) overview network of the full pairwise similarity matrix as a jumping board for localized data exploration. Rather than being based on modified cosine scores, specXplore is based on ms2deepscore pairwise similarities, which can more accurately represent the structural similarities between compounds based on their spectra via a deep-learning-based embedding representation.^3,5,10^ Being trained to predict pairwise structural similarity from spectral data, ms2deepscore in principle allows grouping of similar compounds even if their spectra are dissimilar.^10^ However, ms2deepscore may also introduce a much denser topology. At many reasonable threshold levels, node-link diagram representations of dense matrices tend to become unreadable for the network as a whole.^2^ To facilitate the effective exploration of local neighborhoods, specXplore provides various interactive visualizations, providing views of connectivity surrounding a feature or feature group of interest. Here, partial network drawings and matrix representations play an important role. Localized explorations are combined with the ability to quickly change thresholds to regenerate local views under the new constraints, allowing the careful expansion of neighborhood size for some node of interest. In addition to the network-based representations of local connectivity, specXplore provides means for (a) investigating the raw pairwise similarity matrix directly, (b) investigating the fragmentation overlaps across multiple spectra, and (c) inspecting any joined-in metadata or chemical classifications from within the dashboard.

We will first outline the core components of the tool, their intended usage, and their rationale. This will be followed by illustrative examples on real data and a discussion on the tool in the broader contexts of mass spectral exploratory data analyses.

## Materials and Methods

The specXplore workflow is divided into a Python data importing pipeline and a visual analysis dashboard using dash.^11,12^ The importing part provides data integration and preprocessing functionalities, while the dashboard provides the interactive user interface for data exploration. The tool is available on github under MIT license as a python package that can be downloaded and installed for local use at https://github.com/kevinmildau/specXplore. We will briefly outline the dashboard’s importing pipeline and core visual components.

### Data Importing and Preprocessing

Before data can be opened in the specXplore dashboard, it needs to be processed using the specXplore importing pipeline. The processing workflows and intermediate data structures used by specXplore are built upon a cohort of open-source software Python data science packages, namely, matchms,^13^ MS2Query,^14^ ms2deepscore,^10^ spec2vec,^15^ kmedoids,^16^ Cython,^17^ numpy,^18^ pandas,^19,20^ scikit-learn,^21^ and scipy.^22^ The input data for specXplore spectral data exploration are MS/MS spectral data. LC–MS/MS data preprocessing (i.e., feature detection and MS/MS spectral exporting) is assumed to have been done elsewhere, e.g., using MZMine3, in order to reduce data set size and feature redundancy.^23^ The MS/MS feature data are assumed to be in .mgf (mascot generic format) format, where each entry should have a unique feature identifier, a precursor mass to charge ratio, and spectral data in the form of one or more mass to charge ratio and intensity value tuples. Spectral data are imported into Python using matchms, and basic specXplore data processing is performed (see Supporting Information, Section S2.1).^13^ Spectral data can then be used to initialize a template specXplore object that automatically computes pairwise spectral similarities using three similarity scores: ms2deepscore, modified cosine scores, and spec2vec scores.^3,10,13,15^ For the machine learning scores, pretrained models provided with MS2Query are used.^14^

The central overview of specXplore is based on a t-SNE embedding of the ms2deepscore pairwise similarity matrix serving as the primary similarity score.^21,22,24^ Functionalities are provided for users to test a range of values for the perplexity tuning parameter (usually between 5 and 50) and select an appropriate value. Users will need to balance both high-dimensional distance preservation and network layout qualitative grouping properties.

Similarly, functionalities are provided to construct a range of *k*-medoid clustering-based data subdivisions to complement the t-SNE embeddings. These clusters provide clear local neighborhood groupings that can be otherwise difficult to evaluate given t-SNE’s abstract projection of the similarity matrix.

Finally, to provide the user with chemically informative visual highlighting capacities, MS2Query analog classifications via ClassyFire or NPClassifier, or direct classifications from tools such as Sirius, may be integrated into the specXplore session for visual highlighting capacities (see Supporting Information, Section S2.5).

### SpecXplore Interactive Dashboard

**Figure 1:**
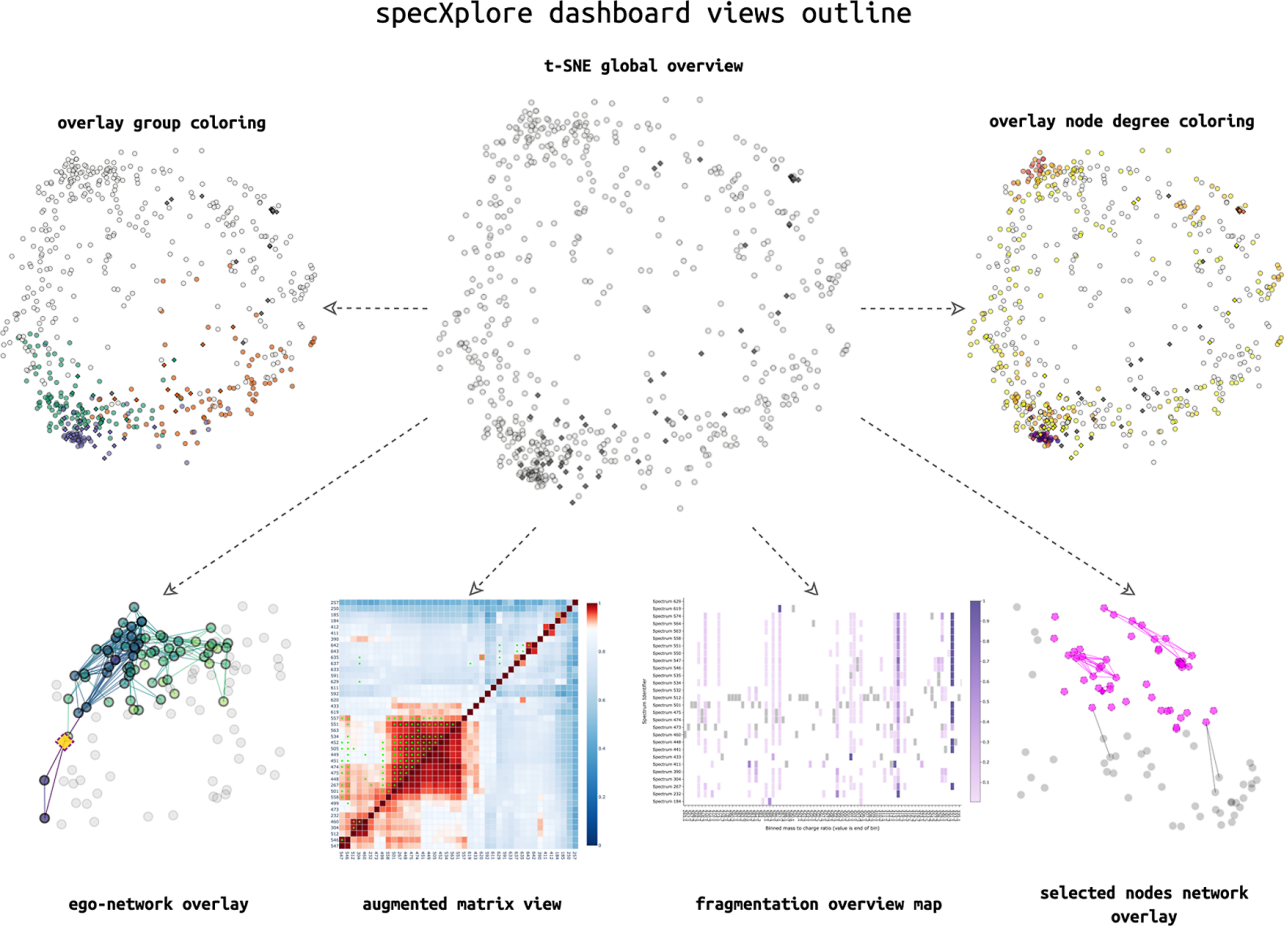
SpecXplore dashboard overview of visual components using wheat data (see the Results section). The central t-SNE overview figure provides a jumping board into three categories of visualizations in the form of color overlays such as group highlighting or node degree visualization, networks overlays such as the ego network or selection network views, and add-on panels such as augmap for quantitative insights into pairwise similarity and fragmap for insights into fragmentation overlaps across selections of features. For a video demonstration, please refer to https://youtu.be/9ZqJAr8wdv8.

SpecXplore provides the user with a variety of views and interactive navigation options (Figure 1).^25^ We envision specXplore’s functionalities to be used in an interactive local exploratory fashion, where the settings and their impact on local neighborhood can be evaluated seamlessly. This local exploration starts at a two-dimensional t-SNE overview figure rendered using dash and dash-cytoscape.^12,26,27^ All other visualizations in specXplore make use of plotly.^28^ In its most basic form, the t-SNE map only contains a node for each feature, and a number of visually highlighted spike-in standard features. Following Shneiderman’s mantra of “Overview first, details on demand”, specXplore allows for the selection of any node, or collection of nodes, for more in-depth analysis in complementary visualization approaches falling into two broad categories: (a) topology overlay views and (b) data details views.^29^

Three different topology-based views are provided. First, to provide insights into the impact of thresholds on the topology, a node degree overlay visualization using a color gradient to represent individual node connectivity levels can be prompted via a button click for the whole t-SNE map (Figure 1 and Supporting Information Figure S2 in Supporting Information Section S3.1.3). This node degree visualization as well as a supporting edge weight distribution plot included in the settings panel follow the principle idea of Willett et al.’s Scented Widgets, providing intuitions about the impact of settings on the topology (see Supporting Information, Section S4.5).^30^ This renders adjusting settings a more informed process.

Second, to provide quantitative insights into the underlying pairwise similarity data of selections of nodes, a heatmap portraying the ms2deepscore pairwise similarity matrix for a selection is provided. Here, the ms2deepscore scores are quantitatively portrayed using a divergent color scale around the current threshold setting, while the exact numeric values are available via mouse-hover panels. This view is called augmap (AUGmented HeatMAP) in specXplore since it also incorporates implied adjacency matrices for modified cosine score and spec2vec score matrices at current threshold settings via additional markers and mouse-hover information, providing a means of comparing adjacency between the scores at current threshold levels.

As a third visualization component, we provide network visualization overlays. For single nodes, specXplore provides so-called ego-network overlays, visualizations suitable for studying a network’s topology relative to a single node.^31,32^ Here, all edges connecting to the selected node, but also all edges connecting to those connections and so forth, are superimposed onto the t-SNE map in line with hop distance settings. This allows the user to explore a branching view of network connectivity emanating from the ego node. For multinode selections, specXplore provides a network view for intragroup connectivity assessments highlighting all edges within a selection of nodes, as well as those connecting outward of the selection.

Finally, specXplore provides a number of data details add-on panels that can be prompted for selections of the nodes. For any group of spectra, the metadata information can be presented as a table, and MS/MS spectrum plots can be generated. Moreover, for pairwise spectral comparison, so-called mirror plots depicting one spectrum on the positive *y* axis and the other on the negative *y* axis are available. However, the latter are not suitable for multispectrum comparison and evaluations of fragmentation overlaps across more than two spectra. To support users with multispectrum comparisons, specXplore provides fragmentation overview heatmaps we refer to as fragmap. To generate a fragmap, binned mass-to-charge ratios are sorted in ascending order and factorized. This allows to (a) reduce the amount of unused white space in each individual spectrum plot by putting the ascending fragments immediately next to each other regardless of mass differences and (b) to separate crowded areas of the mass-to-charge ratio axis into more easily separable pieces. The *y*-axis in the fragmap is used for aligning the different spectra, while a color gradient is used to highlight the fragment intensity. In addition, neutral losses are indicated as constant colored blocks inside the same visualization, providing a rich view of the overlaps in the fragmentation patterns. The fragmap thus provides immediate insights into the spectral overlaps or lack thereof in a single, concise overview. This in turn facilitates assessment of the meaningfulness of local connectivity.

## Results

We illustrate specXplore by applying it on real LC–MS/MS untargeted metabolomics data from two experiments: (a) wheat plants LC–MS/MS data^33,34^ and (b) urine metabolome LC–MS/MS from a polyphenol exposome study.^35^ In both cases, the approach and illustrations are similar. We hence focus on the wheat data here and refer to the supplement for the briefer urine data example (see Supporting Information, Section S3.2). In addition, a video overview of the different views of the tool can be found online at https://youtu.be/9ZqJAr8wdv8. Spectral data from .mgf files was loaded into the preprocessing jupyter notebooks and processed into a specXplore session object (see illustrative example notebooks https://github.com/kevinmildau/specxplore-illustrative-examples). For the wheat data set, the pipeline preprocessing time was less than 15 min (on a MacBook Pro laptop with Apple M1 Pro processor 2021), most of which was spent on local library search via MS2Query. Using the same system, the processing time for the larger urine data set was less than 75 min, whereof approximately 62 min was taken up by MS2Query and 9 min for all pairwise spectral similarity computations.

Upon opening the app and loading the data, the user faces an interactive two-dimensional projection of all spectra created by the t-SNE. Each spectral feature in the data set is represented as either as a circular node or highlighted as darkened diamonds for reference standards in this example. On its own, the t-SNE overview figure is difficult to read. Only a limited view of clustering trends can be observed through denser node regions and positioning of reference standards (Figure 1). This being the case, the t-SNE overview figure still provides an excellent basis for in-depth exploration of the data via interactivity. The most useful top-level exploration features are *k*-medoid clustering at low values of *k* (see Supporting Information, Figure S3A in Supporting Information, Section S3.1.4), chemical classification (see Supporting Information, Figure S3B in Supporting Information, Section S3.1.4), and node degree visualizations (see Supporting Information, Figure S2A in Supporting Information, Section S3.1.3).

Node degree visualization provides an immediate and uncluttered view of the network topology at current threshold levels, providing insights into topological groupings in the data (see Supporting Information, Figure S2A in Supporting Information, Section S3.1.3). In addition, node degree visualizations provide the most straightforward assessment of the impact of threshold changes on local topology while avoiding the computational cost and visual clutter of potentially large numbers of edges (see Supporting Information, Figure S2A,B in Supporting Information Section, S3.1.3).

Color-based highlighting of groupings based on *k*-medoid clustering or chemical ontology predictions can provide additional means of determining the local areas of interest in the t-SNE overview. Here, *k*-medoid clustering provides an edge-threshold-independent means of dividing the feature space into smaller groups while still making use of the pairwise similarity matrix. *K*-medoid clustering tends to show good agreement with both t-SNE projections and topological insights, while it provides a means of determining neighbor sets of nodes of interest (see Supporting Information, Figure S3A). At low *k* values, *k*-medoid clustering tends to produce larger data groupings (see Supporting Information, Figure S3A), higher values of k tend to subdivide the data into smaller, localized clusters often corresponding well to topological connectivity at higher thresholds (see Supporting Information, Figure S4 in Supporting Information, Section S3.1.4). Putative chemical classifications may serve a similar means of prioritizing areas of interest in the t-SNE embedding.

In addition to the use of various color highlighting approaches to detect groupings of interest or achieve a bird’s-eye view of topology, specXplore makes use of edge overlay visualizations granting insights into local node connectivity patterns at adjustable similarity threshold settings (see Supporting Information, Figure S2B in Supporting Information, Section S3.1.3). These views provide insights on what nodes are considered adjacent to one another given the current similarity thresholds. While network views provide simplified views of the similarity relationship between nodes, augmap views provide deeper insights into the similarity matrix and quantitative backbone of all of specXplore’s visualizations (see Supporting Information, Figure S1 in Supporting Information, Section S3.1.2). These quantitative insights can be used to adjust the local threshold settings accordingly or be used as an alternative to network views for small node selections altogether.

**Figure 2:**
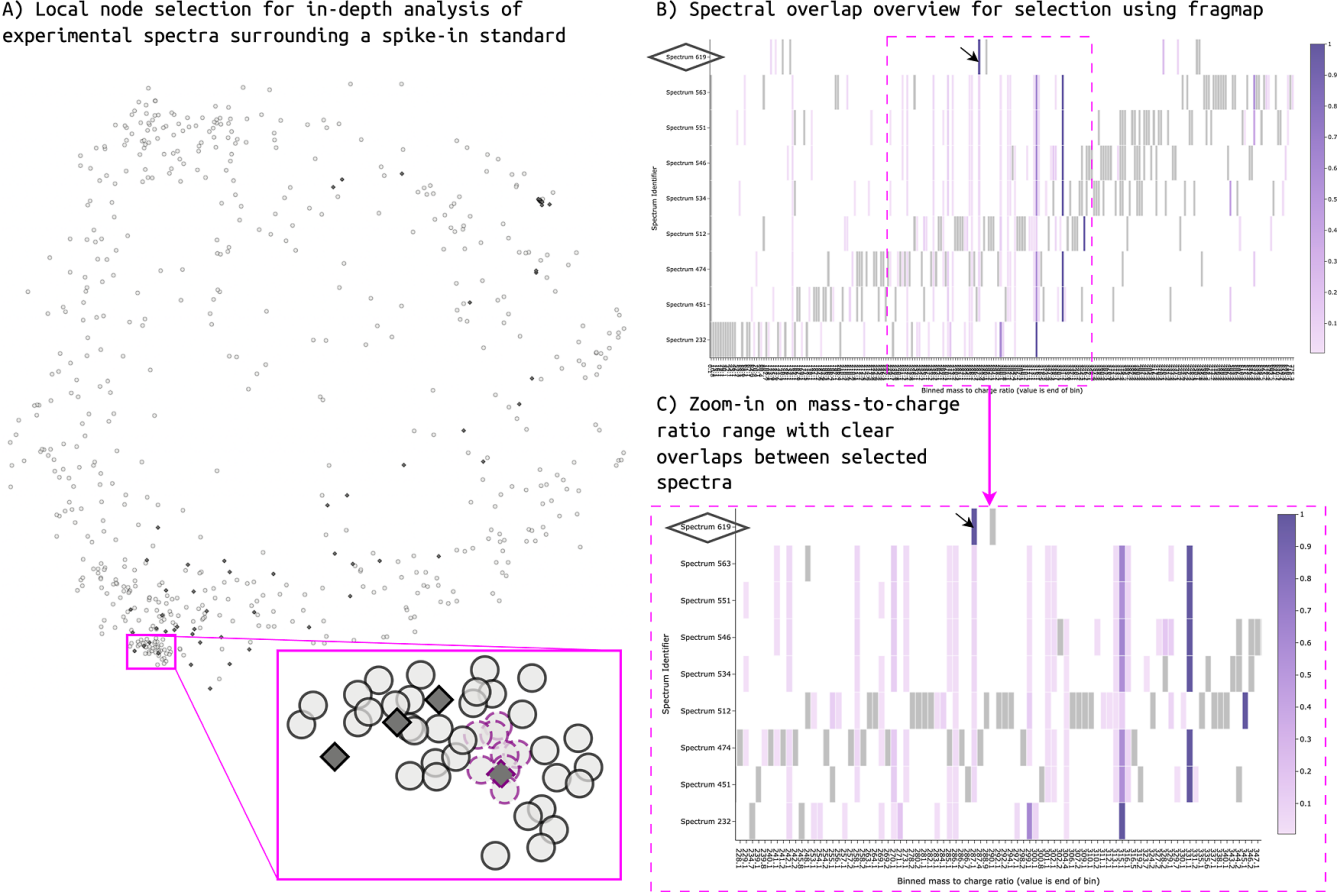
Process of exploring the local environment of a known feature node is illustrated. (A) Procyanidin-A2 reference standard surrounding feature selection in the lower left corner of the t-SNE embedding. The selected nodes are highlighted using magenta outlines. The selected reference standard is highlighted as a dark gray diamond with a dashed selection outline in magenta. (B) Fragmap view of the fragmentation overlap between the spectra selected in A. The reference standard spectrum is of significantly lower complexity than that of the experimental spectra. Only few fragments appear overlapping. However, many fragments are shared across experimental standards in a clear pattern indicating a structural relationship. The reference standard is highlighted on the *y*-axis using an added diamond outline. The highest intensity fragment for the reference standard is further is highlighted using an arrow. (C) Zoom-in of the overlapping area in fragmap view. All except one experimental feature in the local selection share a low relative intensity fragment ion at mass 287.055 that appears to correspond to the most intense fragment in the reference standard.

While specXplore’s global overviews provide insight into the rough patterns of the data, its localized views provide more detailed insights into the possible relationships between the features in the wheat data. There are numerous ways to delve further into the data, given the areas of interest have been found. One sensible approach in specXplore is to explore connectivity around the known reference standards. For instance, exploring the densely connected feature area around the Procyanidin-A2 corresponding feature (Figure 2 A), we can see the characteristic fragment ion overlap as well as the much higher complexity of the experimental spectra (Figure 2 B,C). Such overlaps in fragmentation patterns and implied substructure overlaps alongside local topology and other metadata information may serve as vital starting points for MS/MS structural hypothesis generation and manual annotation efforts. Which features are considered of interest, and how stringent overlaps will need to be to be useful naturally depend on the analysis goals.

## Discussion

We have developed specXplore with two aims in mind: for it to provide a flexible data exploration platform and for it to provide a means of understanding and tailoring network processing settings to the data at hand. In keeping with these aims, specXplore allows the data to be explored interactively with adjustable settings in the well anchored context provided by the t-SNE embedding. In specXplore, there are no rigid subdivisions of the data nor topological parameters to “fix”. We opted for this approach since we think of specXplore as providing an interface to a heterogeneous and complex network of spectral similarities. This network aims to present pairwise structural similarities between spectra via the ms2deepscore model but is impacted by data and model heterogeneity. Here, many different compound classes with different fragmentation behaviors and different model coverages are lumped together into a single, highly heterogeneous network. With data this heterogeneous, local exploration and local setting tailoring seem the most sensible. In practice, specXplore thus requires the user to delve into the network and find localized target groups based on their own criteria of interest and tolerances in spectral and implied structural similarity. While requiring more effort from the user, this also provides them with unprecedented flexibility.

During the development of specXplore, we drew inspiration from two extensions of MN, MolNetEnhancer, and MetGem (see Supporting Information, Figure S6A–C in Supporting Information, Section S4.1).^36−39^ MolNetEnhancer extends MN by providing molecular families with a dominant ms2lda motif classification as a visually highlighteable component of molecular networks in Cytoscape.^40,41^ MetGem extends MN by providing an interlinked network and a t-SNE visualization, providing a means of inspecting the data from two complementary angles at once.^24,37^ Both MolNetEnhancer of MetGem have found use in the field (e.g., refs (42–44) and refs (45–47)) and highlight the potential in extending and tailoring the MN workflows.

We also drew inspiration from the network visualization tool EdgeMaps (see Supporting Information, Figure S6D in Supporting Information, Section S4.1).^39,48^ In EdgeMaps, a complex and dense network is embedded in a two-dimensional projection of node similarities, and directed edges between any interconnected nodes are visualized only upon interactive demand.

SpecXplore makes use of the embedding approach of MetGem and EdgeMaps to create a similarity preserving layout and makes use of interactive prompting as in EdgeMaps to highlight the local topological relationships. In addition, chemical space prioritization is facilitated through chemical-classification-based node coloring as done in MolNetEnhancer.

Naturally, as a tool for spectral data exploration, specXplore draws inspiration from Network Annotation Propagation, be it automatic or manual, where we designate the primary aim of specXplore to be the exploration of the local topology with the goal of structural hypothesis propagation and spectral cross comparison.^8,49,50^ Combining elements of all these approaches, specXplore is a uniquely flexible data exploration approach for mass spectral data that covers a broad range of complex network visualization tasks.^51^

### Impact of Settings in Traditional Molecular Networking and SpecXplore

MN, as hosted on GNPS, provides an interesting and successful framework for topology-based mass spectral exploratory data analysis (EDA).^4^ EDA is characteristically dynamic and flexible, yet the analysis approach and settings may have a strong impact on how the data is viewed and used. The primary topological settings in MN are (a) the pairwise similarity thresholds used as well as corresponding minimum fragmentation overlaps, (b) top-K neighbor limits on individual nodes, and (c) maximal molecular family sizes. Additionally, one can consider the choice of the modified cosine score (or spec2vec scores) as the underlying metric a setting. In the GNPS workflow, these settings are used to create a subdivision of the spectral data into disjoint groupings that are visualized separately as subnetworks. This subdivision is possible because of the general connection sparsity encouraged by the settings: modified cosine similarity matrices will be comparatibely sparse, while typical default thresholds of 0.7 will lead to even sparser adjacency matrices. In addition, top-K limitations on the number of neighbors for each node can limit the scope of hub nodes and reduce cross-network connectivity. Thus, the combination of settings and visualization approaches are tailored to accommodate sparse representations and not dense networks. A feature of MN is thus that that molecular families represent groups of high spectral similarity with no visible links to other families. Missing connections between nodes and clusters through restrictive settings, but in rarer instances also hard to decipher hairball molecular networks through too liberal settings, may be encountered.

With settings being as impactful, it is hence a key issue that GNPS reruns with different settings are slow, while comparisons of runs from one to another are nontrivial. Indeed, MN produces disjoint molecular families to be analyzed separately, while reruns with different settings produce different molecular families in size, composition, and natural ordering. In addition, individual molecular families make use of randomized force-directed layouts, leading to possibly different node positioning in each run and family. This means that the preservation of any kind of mental map of the different runs and how they compare against one another are difficult. While the speed bottleneck of traditional MN on GNPS can be partly overcome with tools such as MetGem or MZMine, which allow fast local reruns separating data processing from networking settings, the comparison of different runs to one another still remains difficult.^23,37^

The comparability issue is addressed in specXplore via its local subnetwork and information on demand approach. The use of fixed t-SNE coordinates as layout allows the user to create a mental map of the data, as well as allows them to study the impact of on-the-fly changeable network settings on their requested local views easily against the thus provided visual anchor-point.

In addition to fundamental visual design differences, specXplore makes use of the ms2deepscore model for generating the pairwise similarity matrix underlying its visualizations.^10^ This model is used as it provides better capabilities for linking spectra with stronger fragmentation differences, albeit at the cost of denser networks that are more challenging to visualize.^10^ However, since ms2deepscore is a machine learning model it can only be expected to work well for spectra and metabolites well covered by or related to its training data. To accommodate this deficit at least in part, specXplore provides the augmap views granting insights into the differences between ms2deepscore, modified cosine scores, and spec2vec scores on the same data.

The specXplore dashboard has been shown in our illustrative examples to work well for the wheat data and the urine data sets containing <1000 and <4000 features each after processing. For these smaller, processed data sets, specXplore works provides broad flexibility and interconnected visual analysis features. We expect that the dashboard will scale well to 5000 features but face difficulties for larger data sets and liberal settings. Network representations may quickly become visually overwhelming or computationally demanding to process and render. The feature-rich and liberal settings approach of specXplore does not lend itself to repository scale analyses.

### Dense Networks and Network Layout Choice

Since specXplore contains a large network analysis component, the choice of feature positioning in its general overview is in part a question of network layout choice. Laying out dense networks is a difficult task often addressed using force-directed layout algorithms owing to their computational tractabiity.^52−55^ However, the latter do not scale well to large and dense networks and tend to produce hard-to-read or unintelligable networks rendition.^55,56^ This is particularly because of often created dense “hairballs” of nodes and edges, as well as edge-crossings.^57−59^ In specXplore, this is addressed by a combination of latent variable space embedding of nodes with interactively triggered partial network drawings.^37,39,60,61^ Here, the latent variable embedding serves as a jumping board for localized network explorations. This approach is in line with Shneiderman’s mantra of “Overview first; details on demand”.^29^ Dense hairball visualizations or edges traversing the whole t-SNE embedding are avoided by only visualizing edges on demand, using stringent user-modifiable edge filter settings.

Alternative approaches available to dealing with poor readibilty make use of summarization.^62^ The nodes can be hierarchically aggregated, or the edges can be bundled.^63,64^ Such approaches however tend to alter the preceived relationships within the graph and ultimately require interactivity such as hypernode expansion or semantic zooming to allow insights into the various levels of granularity.^65−67^ Hence, no matter the approach taken, some form of interactive data visualization is needed to handle dense networks. The approach used in specXplore aims to only minimally alter the perceived topology of the network and provide intuitive and easily understandable forms of interaction via interactive overlays.

It should be noted, however, that the use of t-SNE as the layout approach in specXplore does not come without disadvantages. Embeddings produced by t-SNE are built to preserve local neighborhoods in the high-dimensional space in their projection to a two-dimensional space.^24^ This focus may lead to difficulties in interpretation of t-SNE results as neglect of global similarity preservation may render cluster proximity a poor indicator of cluster similarity.^68^ Alternative approaches such as UMAP or PaCMAP may be more capable at preserving global or even global and local trends.^69−71^ However, recent research shows that t-SNE artifacts may be avoided with very careful method tuning.^68,72^ We note that t-SNE’s local focus works well within specXplore as it tends to group together high-similarity nodes, while global-similarity distortions are partially offset by the use of complementary visualization overlays such as network representations which afford a view of effective connectivity between groupings at given threshold levels.

### *K*-Medoid Clustering in SpecXplore

The t-SNE embedding overview panel of specXplore provides the user with an abstract and condensed representation of the pairwise similarity matrix lacking a clear node grouping structure. To alleviate this problem, specXplore provides complementary *k*-medoid clustering color overlays, where clustering at various values of k provides quick glances at local neighborhoods in the t-SNE overview. The *k*-medoid clustering algorithm is closely related to *k*-means clustering, where *k*-medoid omits the necessity of computing some form of centroid against which to measure distances, instead making use of the median distanced observation within a cluster, i.e., the medoid, as the reference against which to measure distances.^73^*K*-medoid clustering thus has a number of advantages within specXplore: (a) any arbitrary distance measure may be used with *k*-medoid clustering, including ms2deepscore itself, (b) making use of medoids circumvents the need of defining centroids, as well as any associated needs for recomputing distances from the latter making it computationally cheap, and (c) since *k*-medoid operates directly on the similarity matrix its cluster assignments are unaffected by t-SNE projection artifacts.

We considered making use of hierarchical clustering with medoid linkage for maintaining cluster consistency across levels of granularity.^74^ Clusters being hierarchically subdivided into subclusters, which in turn are further subdivided, and so on, would provide a mental map advantage when exploring different granularity levels. For *k*-medoid clustering, no such agreement across different values of *K* is enforced, and hence, cluster assignments may vary across settings of *k* sometimes grouping features together and sometimes not. However, due to both lacking implementation availability in Python and suboptimality of the hierarchically constrained clusters at any level of *k*, we opted to use *k*-medoid clustering only.^74^ In specXplore, we make use of *k*-medoid clustering as an assistive grouping approach rather than an end-point. Hence, different values of k are used to provide variable granularity groupings to be further explored in the general t-SNE embedding and using other complementary views such as partial network drawings. More work on measuring and comparing optimality would be needed to provide users with stronger guidelines for automatic cluster tuning.

## Conclusions

SpecXplore provides a means of interactively slicing into the complete matrix of pairwise spectral similarities via adjustable settings and complementary views. Exploration of the data in this way provides a means of determining spectral neighborhoods of interest and to assist direct and indirect network annotation propagation. In addition, specXplore exposes the impact of topological filtering settings on effective topology and local neighborhood contexts. Being based on ms2deepscore, it further allows for state-of-the-art similarity scoring that reflects structural similarities more closely. This in turn opens up opportunities for finding similarities missed by traditional scoring approaches. SpecXplore thus provides users with flexible state-of-the-art data exploration platform. Future works for specXplore we are considering are (a) providing an online hosted version for more straightforward accessibility and (b) an integration with statistical testing results for more effective prioritization leveraging experimental designs (such as in FERMO^75^).

## Supporting Information Available

Supplementary notes and details regarding the materials discussed in the manuscript (PDF)
